# Protect European green agricultural policies for future food security

**DOI:** 10.1038/s43247-022-00550-2

**Published:** 2022-09-20

**Authors:** Manuel B. Morales, Mario Díaz, David Giralt, Francesc Sardà-Palomera, Juan Traba, François Mougeot, David Serrano, Santi Mañosa, Sabrina Gaba, Francisco Moreira, Tomas Pärt, Elena D. Concepción, Rocío Tarjuelo, Beatriz Arroyo, Gerard Bota

**Affiliations:** 1 Departamento de Ecología, Universidad Autónoma de Madrid, Madrid, Spain; 2 Centro de Investigación en Biodiversidad y Cambio Global, Universidad Autónoma de Madrid, Madrid, Spain; 3 Museo Nacional de Ciencias Naturales, CSIC, Madrid, Spain; 4 Conservation Biology Group, Landscape Dynamics and Biodiversity Program, Conservation Biology Group (GBiC), Forest Science and Technology Centre of Catalonia (CTFC), Solsona, Spain; 5 Instituto de Investigación en Recursos Cinegéticos (CSIC-UCLM-JCCM), Ciudad Real, Spain; 6 Estación Biológica de Doñana, CSIC, Sevilla, Spain; 7 Departament de Biologia Evolutiva, Ecologia i Ciències Ambientals, Facultat de Biologia, Universitat de Barcelona, Barcelona, Spain; 8 Institut de Recerca de la Biodiversitat (IRBio), Universitat de Barcelona, Barcelona, Spain; 9 USC 1339 Centre d’Etudes Biologiques de Chizé, INRAE, CNRS & Université de La Rochelle, F-79360 Villiers-en-Bois, France; 10 UMR 7372 Centre d’Etudes Biologiques de Chizé, CNRS & Université de La Rochelle, F-79360 Villiers-en-Bois, France; 11 CIBIO/InBio–University of Porto and Institute of Agronomy–University of Lisbon, Lisbon, Portugal; 12 Swedish University of Agricultural Sciences, Uppsala, Sweden; 13 Sustainable Forest Management Research Institute (iuFOR), Universidad de Valladolid & INIA, Valladolid, Spain

**Keywords:** Agroecology, Environmental social sciences

## Abstract

In their Comment in @CommsEarth, Manuel Morales and colleagues argue that we must act now to protect green agricultural policies in the EU to ensure food security in the future.

## Teaser

European green agricultural policies have been relaxed to allow cultivation of fallow land to produce animal feed and meet shortfalls in exports from Ukraine and Russia. However, conversion of semi-natural habitats will disproportionately impact long term biodiversity and food security.

## Introduction

The European Union’s new (2023–2027) Common Agricultural Policy (CAP) aims to reverse current environmental degradation and biodiversity declines in European farmland^1^ through the achievement of three green objectives: contribute to climate change mitigation, support efficient natural resource management, and reverse biodiversity loss^2,3^. Following the outbreak of war in Ukraine, the European Commission proposed a series of short and medium-term relaxations to CAP’s environmental commitments to offset expected shortages in grain imports and enhance food security^4^.

Here, we argue that policy changes to allow cultivation of fallow land will disproportionately impact biodiversity and support further intensification of livestock production. Thus, ultimately, these changes in policy may sacrifice long term biodiversity and agricultural sustainability in Europe, in favour of modest increases in current agricultural production and alleged improvements of food security.

### A catalyst for reversing green policies

**Fig. 1:**
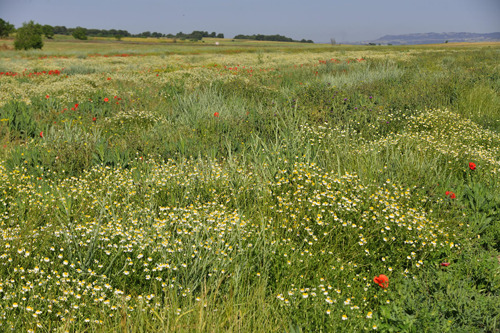
Arable field left fallow and allowed to develop a grassy vegetation cover. Under non-intensive management, fallow areas become a genuine semi-natural habitat, key for the conservation of farmland biodiversity. Credit: Jordi Bas, taken in the cereal steppes of the Lleida plain (Catalonia, Spain).

Russia and Ukraine are world-leading producers and exporters of cereal and fodder production (notably, oleo-proteaginous crops)^5^. The Ukraine war and international sanctions on Russia are threatening the import of these products to the EU. Ukrainian winter cereal, maize and sunflower production is expected to decrease by 20–30%, at least during the 2022–2023 season, and similar reductions in Russian exports are also expected^5^. Therefore agro-industry lobbies and farmers’ organisations in Brussels, some political parties in the European parliament and some countries’ administrations perceive a need to increase agricultural production^6^ and, as a means to offset expected shortages, are pressing to relax or remove CAP’s environmental commitments. Mechanisms supporting these commitments include enhanced conditionality (compulsory for all farmers receiving subsidies), voluntary measures of Rural Development Programmes (i.e. agri-environment-climate-measures) and Greening measures (crop diversification, maintenance of permanent grasslands and promotion of Ecological Focus Areas). A call made to mobilise all relevant international groups during the informal meeting held on 2 March 2022 by Member States’ agriculture and food ministers, with the exception of Denmark, Germany and Italy, may reflect such pressure^6^. Indeed, the European Commission has finally proposed a series of “short- and medium-term actions to enhance global food security and to support farmers and consumers in the EU”^4^. In regard to land-use, actions refer to the cultivation of fallows, which are protected by green payments for keeping land in good agricultural and environmental conditions and, adequately managed (both long-term and annual), support high levels of biodiversity and ecosystem services^7^ (Fig. 1). More precisely, the European Commission proposes that “*To enlarge the EU’s production capacity, the Commission has today adopted an implementing act to exceptionally and temporarily allow Member States to derogate from certain greening obligations. In particular, they may allow for production of any crops for food and feed on fallow land that is part of Ecological Focus Areas in 2022, while maintaining the full level of the greening payment*”^4^. This measure was recently extended for 2023.

### Considering food sovereignty

However, the FAO does not draw the same conclusions about the possible world impacts of the conflict and recommends finding alternative suppliers, instead suggesting using existing food stocks, diversifying domestic crops and reducing fertiliser dependence and food waste as mechanisms to help guarantee Europe’s food supplies and sovereignty^5^. Even the European Commission, while acknowledging the vulnerability of European farmers to animal feed import shortages and increased costs, clearly stated that food supply is not at risk in the EU^4^. Indeed, EU-based production supplies 79% of the feed proteins consumed in European livestock farming, 90% of feed cereals and 93% of other products such as Dried Distillers’ Grains and Solubles or beet pulp^8^. In 2020, the EU was completely self-sufficient with respect to dairy products, pork, beef, veal, poultry, and cereals. It remained the largest global exporter of agri-food products, in spite of the COVID-19 pandemic^8^.

### Counterproductive policies

Any increase in production from cultivating fallow land will therefore likely be used to feed intensively reared livestock and sustain cattle feed exports. Supporting the increasing trend of feed exports and industrial intensive livestock farming does not align with the EU’s Green Deal due to the negative impacts on air, soil and water quality^8–10^. In addition, cultivating fallow land to support intensive livestock-based agriculture will undermine the EU’s Farm-to-Fork strategy and CAP’s ‘Food and Health’ objective of reducing meat consumption to favour a more sustainable and healthier diet among European consumers^2,11^. Encouraging the growth of intensive livestock farming through enabling cultivation of fallow lands will increase environmental damage, biodiversity loss and public health risks. Thus, the recent relaxations of the new CAP compromise several of its fundamental objectives, along with those of other elements of the Green Deal, such as the EU’s Nature Restoration Law^2,9,12^.

The duration of the war in Ukraine and its effects on provision of raw materials to Europe is hard to foresee. We acknowledge the uncertainties and input costs faced by farmers but calls for further agricultural intensification may be largely unjustified at this stage. Specifically, cultivating semi-natural habitats like long-term or unploughed annual fallows will have serious environmental costs, including an increase in pesticide and fertiliser application, since fallows often occupy less productive land^13^. Even a moderate increase in food production at the expense of the semi-natural habitats remaining in farmland landscapes (field margins, grasslands, and fallow land), which support most of Europe’s farmland biodiversity and its associated ecosystem services^14^, will seriously damage farmland biodiversity and sustainability in European agricultural landscapes^3,15^. For example, a comprehensive study carried out on 169 farms across 10 European countries showed that semi-natural habitats, including fallows, occupied 23% of the land but hosted 49% of vascular plant, earthworm, spider, and wild bee species; a 10% decrease of these habitats if reclaimed for food production would cause exponential decreases in biodiversity, but only moderate linear increases in production^15^. Furthermore, the loss of semi-natural habitats in arable systems, fallows among them, would negatively affect arthropod functional diversity and the ecosystem services it supports, which may affect agriculture production^14^.

### Sustainable alternatives

There are alternatives to cultivating semi-natural habitats that may (and need to) be assessed to achieve a more strategic European agricultural policy able to meet food demands while maintaining the sustainability principles and improvements of the food-production chain sought by the Farm-to-Fork strategy. Proposals include agro-ecological approaches to increase production through the enhancement of ecosystem services such as pollination and biological control^16–18^, adjusting the amount of cultivated surface in relation to landscape structure and composition^19^, or relocating crops that are more in demand to areas where production is optimal without increasing the total cultivated area^20^.

After decades of costly implementation and reforms of agricultural and conservation policies^1^, the EU is at risk of engaging in a hasty and misguided strategy on food production jeopardising the green transition^13^. As an alternative to such a ‘business as usual’ reaction, the EU has now the opportunity to consolidate the mentioned environmental and social objectives of the new CAP^2,3^. A more sustainable agriculture, resilient to food supply crises (present and future), should be based on ecological functionality of farmland, which ultimately depends on the conservation of its biodiversity^16^, along with measures to counter climate change. Responses to this and other challenges on the new CAP should be assessed with a long-term perspective and based on robust scientific evidence before undermining its environmental ambitions^3,13^.

## References

[1] Emmerson M (2016). How agricultural intensification affects biodiversity and ecosystem services. Adv. Ecol. Res..

[2] European Commission. The new common agricultural policy: 2023-27. europa.eu (2022).

[3] Pe’er G (2022). How can the European Common Agricultural Policy help halt biodiversity loss? Recommendations by over 300 experts. Cons. Lett..

[4] European Commission, Brussels. 133 final. Safeguarding food security and reinforcing the resilience of food systems. https://ec.europa.eu/info/sites/default/files/food-farming-fisheries/key_policies/documents/safeguarding-food-security-reinforcing-resilience-food-systems.pdf (2022).

[5] Food and Agriculture Organization of the United Nations. *Information Note, 10 June 2022 Update. The Importance of Ukraine and the Russian Federation for Global Agricultural Markets and the Risks Associated with the Current Conflict* (FAO, 2022).

[6] European Council. Informal video conference of agriculture ministers, 2 March 2022 European Council 2022 Informal video conference of agriculture ministers, 2 March 2022.

[7] Tarjuelo R, Margalida A, Mougeot F (2020). Changing the fallow paradigm: a win–win strategy for the post 2020 Common Agricultural Policy to halt farmland bird declines. J. Appl. Ecol..

[8] European Feed Manufacturers’ Federation. *Feed and Food 2021.**FEFAC. Feed & Food Statistical Yearbook 2021* (European Feed Manufacturers’ Federation, 2021).

[9] Kraham, S. J. in *International Farm Animal, Wildlife and Food Safety Law* (eds. Steier, G. & Patel, K. K.) 3–40 (Springer, 2017)

[10] Smit LAM, Heederik D (2017). Impacts of intensive livestock production on human health in densely populated regions. GeoHealth.

[11] European Commission. Farm to fork strategy. europa.eu (2022).

[12] European Commission. Green deal: pioneering proposals to restore Europe’s nature by 2050 and halve pesticide use by 2030. https://ec.europa.eu/commission/presscorner/detail/en/ip_22_3746 (2022).

[13] Strange N (2022). Policy responses to the Ukraine crisis threaten European biodiversity. Nat. Ecol. Evol..

[14] Martin E (2019). The interplay of landscape composition and configuration: new pathways to manage functional biodiversity and agro-ecosystem services across Europe. Ecol. Lett..

[15] Jeanneret P (2021). An increase in food production in Europe could dramatically affect farmland biodiversity. Commun. Earth Environ..

[16] Montoya D, Gaba S, de Mazancourt C, Bretagnolle V, Loreau M (2020). Reconciling biodiversity conservation, food production and farmers’ demand in agricultural landscapes. Ecol. Model..

[17] Catarino R, Bretagnolle V, Perrot T, Vialloux F, Gaba S (2019). Bee pollination outperforms pesticides for oilseed crop production and profitability. Proc. R. Soc. B.

[18] Catarino R, Gaba S, Bretagnolle V (2019). Experimental and empirical evidence shows that reducing weed control in winter cereal fields is a viable strategy for farmers. Sci. Rep..

[19] Wretenberg J, Pärt T, Berg Å (2010). Changes in local species richness of farmland birds in relation to land-use changes and landscape structure. Biol. Cons..

[20] Beyer, R. M., Hua, F., Marin, P. A., Manica, A. & Rademacher, T. Relocating croplands could drastically reduce the environmental impacts of global food production. *Commun. Earth Environ*. **3**, 49 (2022).

